# SynCraft: an integrated web server for ADMET-aware retrosynthesis and molecular design

**DOI:** 10.1093/nar/gkag463

**Published:** 2026-05-14

**Authors:** Qahtan Adnan Aljanabi, Zhijian Huang, Jinmiao Song, Lei Deng

**Affiliations:** School of Computer Science and Engineering, Central South University, Changsha 410083, China; School of Computer Science and Engineering, Central South University, Changsha 410083, China; School of Software, Xinjiang University, Urumqi 830046, China; School of Computer Science and Engineering, Central South University, Changsha 410083, China; School of Software, Xinjiang University, Urumqi 830046, China

## Abstract

The efficient design of drug candidates requires simultaneous consideration of synthetic feasibility and pharmacokinetic safety. However, current computational tools typically address retrosynthetic planning and ADMET evaluation as separate tasks, requiring users to manually transfer data between platforms and evaluate candidate molecules in a sequential and time-consuming manner. Here, we present SynCraft, a web server that integrates template-based retrosynthesis with real-time ADMET evaluation within a unified workflow. SynCraft accepts molecular input via SMILES, interactive drawing, or file upload, and performs automated multi-step retrosynthetic planning while simultaneously assessing the safety and drug-likeness of all intermediate compounds. ADMET properties are predicted using a multi-view learning framework, and potentially hazardous intermediates are automatically highlighted with intuitive, colour-coded warnings during route exploration. The server further provides integrated analysis tools, including synthetic accessibility and complexity scoring, drug-likeness evaluation, and interactive 2D/3D visualization, enabling users to seamlessly transition from route generation to detailed molecular assessment. Results are presented through an interactive interface that allows comparison of alternative synthetic routes, with downloadable outputs in standard formats. In a case study of imatinib, SynCraft automatically identifies genotoxic intermediates that are typically overlooked in conventional retrosynthesis workflows, demonstrating its practical utility for safety-aware route design. The web server is freely accessible at https://syncraft.denglab.org.

## Graphical Abstract

**Graphical Abstract:**
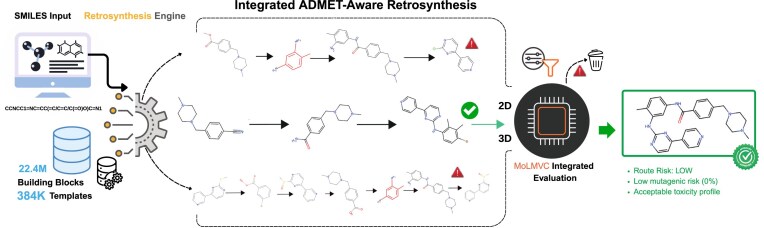

## Introduction

The transition from early hit identification to a viable clinical candidate remains one of the most challenging stages of pharmaceutical development, often referred to as the valley of death in drug discovery [1]. Although modern virtual screening can explore chemical spaces exceeding $10^{60}$ molecules, the subsequent hit-to-lead and lead optimization stages remain constrained by a fundamental synthesis–safety disconnect [2]. Approximately 40% of preclinical candidates ultimately fail because of unfavourable ADMET properties that are only detected late in the development process [3, 4]. Despite advances in generative artificial intelligence and high-throughput screening, these attrition rates remain high, in part because synthetic feasibility and biological safety are typically evaluated in separate, sequential computational workflows. Computer-aided synthesis planning (CASP) tools such as AiZynthFinder and ASKCOS can efficiently generate plausible synthetic routes for complex molecules [5, 6]. However, these systems focus primarily on chemical feasibility and do not assess the toxicological properties of intermediate compounds generated during route exploration. In contrast, ADMET prediction platforms such as SwissADME and ADMETlab 3.0 provide comprehensive pharmacokinetic and toxicity assessments but are typically applied post-hoc to final candidates rather than to intermediates during route design [7, 8]. As a result, medicinal chemists must manually connect these tools within an iterative design–make–test–analyse (DMTA) cycle. This separation can be particularly problematic when hazardous intermediates arise during synthetic planning. For example, studies have shown that a large fraction of common synthetic building blocks possess mutagenic potential [9, 10], and insufficient early detection of genotoxic impurities has contributed to major pharmaceutical contamination incidents such as the valsartan and ranitidine recalls [11, 12].

Another limitation of many existing computational tools is their reliance on simplified 1D or 2D molecular representations, which may fail to capture stereochemical and three-dimensional structural features that influence biological activity and toxicity [13, 14].

To address these challenges, we developed SynCraft, a freely accessible web server that integrates template-based retrosynthesis with real-time ADMET safety evaluation within a unified workflow. SynCraft combines a large library of 384,512 reaction templates derived from USPTO data with the MolMVC multi-view contrastive learning framework for molecular property prediction [13, 15]. By incorporating ADMET prediction directly into the retrosynthetic search process, SynCraft enables early identification of potentially hazardous intermediates and guides the discovery of synthetic routes that are both chemically feasible and toxicologically acceptable.

## Materials and methods

### Overview of SynCraft

SynCraft is a web server designed to integrate two previously distinct computational capabilities into a single, automated pipeline. The core components are as follows: the retrosynthesis engine utilizes a template-based BFS search built on RDKit, RDChiral, and RXNMapper, with a template library extracted from the USPTO-MIT dataset and augmented by the version used in Retro* [15]. Complementing this, the ADMET prediction module is powered by MolMVC, a multi-view contrastive learning framework published by our group [13]. By integrating multiple molecular representations, MolMVC provides robust predictions of toxicity and pharmacokinetic properties for all intermediates generated during the retrosynthetic search.

The novel contribution of SynCraft is threefold: (i) the automated pipeline integration of ADMET prediction with retrosynthesis, ensuring that every generated intermediate is evaluated without manual intervention; (ii) the integrated ADMET flagging mechanism that uses MolMVC predictions to automatically assess every intermediate across all generated routes, highlighting hazardous compounds with colour-coded warnings so that chemists can immediately identify and avoid unsafe pathways—an outcome typically requiring tedious manual cross-referencing in sequential workflows; and (iii) a comprehensive analysis ecosystem within a unified web interface. Beyond retrosynthetic planning, SynCraft integrates an interactive suite for deep molecular analysis, including real-time **s**ynthetic accessibility (SA) and complexity (SC) scoring, 3D conformer visualization, and multi-parameter drug-likeness evaluation (QED and Lipinski compliance). This consolidated environment eliminates the manual file-export, data-reformatting, and tool-switching overhead inherent in existing sequential workflows, allowing chemists to transition seamlessly from route discovery to detailed lead optimization in a single session. A systematic comparison with existing platforms is shown in Table 1.

**Table 1.** Comparison of SynCraft with established retrosynthesis and ADMET platforms. No existing tool combines multi-step retrosynthetic planning with real-time safety evaluation of synthetic intermediates. AiZynthFinder and ASKCOS perform no molecular property assessment; SwissADME and ADMETlab 3.0 evaluate only user-submitted molecules, not intermediates generated during route exploration. Symbols: $\checkmark$ = supported; $\times$ = not supported; N/A = not applicable

Feature	SynCraft	AiZynthFinder [5]	ASKCOS [6]	SwissADME [7]	ADMETlab 3.0 [8]
Primary function	**Unified**	Retrosynthesis	Retrosynthesis	ADMET	ADMET
	**Retrosyn.+ADMET**	only	only	only	only
Integrated pipeline	$\checkmark$	$\times$	$\times$	N/A	N/A
Retrosynthesis	$\checkmark$	$\checkmark$	$\checkmark$	$\times$	$\times$
Toxicity prediction	$\checkmark$	$\times$	$\times$	$\times$	$\checkmark$
Real-time flagging	$\checkmark$	$\times$	$\times$	$\times$	$\times$
Intermediate evaluation	**All intermediates**	$\times$	$\times$	Final molecule only	Final molecule only
Molecular representation	**1D + 2D + 3D**	1D + 2D	1D + 2D	1D + 2D	1D + 2D
No registration	$\checkmark$	$\checkmark$	$\checkmark$	$\checkmark$	$\checkmark$

### The SynCraft algorithm

SynCraft operates through five integrated modules: (1) a Molecular Editor featuring a Ketcher-based interface for interactive structure sketching; (2) a Retrosynthesis and ADMET Planner that generates synthetic pathways and automatically evaluates intermediate safety; (3) a Synthetic Accessibility Scorer referencing 22.4 million commercial building blocks; and (4) a Visualization and Analysis Suite providing dedicated 2D/3D molecular rendering and detailed property reporting; (5) aFormat Conversion Utility for interconverting chemical formats, including SMILES, MOL, and SDF.

#### Template library and retrosynthesis algorithm

Templates were drawn from two sources: (i) USPTO-MIT and (ii) templates from the USPTO-MIT dataset used by Retro* [15]. The combined library contains 384,512 unique templates ($n=3{,}210$ USPTO; $n=381{,}302$ Retro*), interleaved by descending frequency rank and scored via exponential decay:


(1) \begin{eqnarray*}
s_i = \exp (-i/100) \cdot Z
\end{eqnarray*}

where $i$ is the position in the interleaved ranked list and $Z$ normalizes the distribution. The decay constant of 100 was selected empirically over the range 50–500 on a 500-molecule validation set; values below 50 over-weight rare templates causing search instability, while values above 500 approach uniform weighting with increased search time. The selected value of 100 gave the best balance of solve rate and runtime (Supplementary Section S1.4).

Retrosynthetic planning uses BFS with InChIKey cycle detection, capped at six steps. BFS was selected over MCTS for deterministic reproducibility and predictable depth-bounded runtime across diverse query molecules. The depth cap was selected based on a dedicated depth-calibration experiment showing that 94.3% of molecules were solved within six steps. This 94.3% value is specific to the depth-calibration set and is not directly comparable to the 71% solve rate of the primary benchmark, which uses a separate held-out evaluation set under full production settings. Extending to eight steps gained only 1.8% in solve rate at a 4.2-fold runtime increase (Supplementary Section S1.7). Proposed disconnections are validated by a complexity-aware heuristic reducing candidate space by $\sim$70% prior to deep evaluation. The multi-objective Route Score is:


(2) \begin{eqnarray*}
\text{Route Score} &=& w_1/\mathrm{SA}_{\mathrm{avg}} + w_2/\mathrm{SC}_{\mathrm{avg}} \\&& + w_3 \cdot \mathrm{ADMET}_{\mathrm{min}} + w_4 \cdot e^{-n/3}
\end{eqnarray*}

where $w_1\!=\!0.3$, $w_2\!=\!0.2$, $w_3\!=\!0.3$, $w_4\!=\!0.2$. Weights were optimized on a held-out set of 200 ChEMBL molecules with known synthesis routes by maximizing the rank correlation between Route Score and expert chemist preference ratings (Spearman $\rho \!=\!0.71$, $p$ $< $ 0.001; Supplementary Section S1.6). Sensitivity analysis showed that varying individual weights $\pm$0.1 changed the top-ranked route in fewer than 12% of cases, confirming robustness to the exact weight values (Supplementary Table S2). The search terminates at the 22.4-million building-block database (ZINC15 and eMolecules) (Supplementary Section S1.8).

#### ADMET prediction: MolMVC framework

SynCraft’s safety engine deploys MolMVC [13], fusing three molecular views: a **1D** 6-layer Transformer on ESPF fingerprints; a **2D** GIN + Graph Transformer on molecular topology; and a **3D** SchNet-based Graph Transformer on ETKDG/MMFF94 conformers. Views are aligned by Adaptive Multi-View Contrastive Loss (AMCLoss) at local and global levels. Average 3D conformer generation time is 8 ms (MW $< $ 300), 23 ms (MW 300–500), and 67 ms (MW $> $ 500) per molecule, measured on the benchmark hardware; molecules with $> $15 rotatable bonds may require up to 180 ms (Supplementary Section S3.2). Inference is executed on GPU to leverage batch parallelism, achieving a throughput of 10 000 evaluations per hour (Supplementary Section S1.11).

SA scores are calculated using TwistDAN, developed in our previous work [16], which utilizes a domain-adaptive synthetic accessibility predictor employing gradient reversal domain adaptation and SELFIES-based augmentation to produce robust accessibility scores across diverse chemical spaces, outperforming the conventional Ertl and Schuffenhauer method [17] on structurally diverse benchmarks. SC scores use the SCScore model [18]. RDKit computes 200+ 2D descriptors; ETKDG with MMFF94 optimisation yields 3D shape metrics.

Lipinski Rule of Five compliance [19] is assessed for all intermediates, with $\ge$2 violations triggering visual warnings in the user interface. It should be noted that zero violations is the strict criterion used for benchmarking in (Supplementary Table S5) whereas the $\ge$2-violation threshold is reserved for real-time interface alerts. As a holistic complement to binary assessment, the Quantitative Estimate of Drug-likeness (QED) [20] is computed for every intermediate. By aggregating eight physicochemical properties through continuous desirability functions, QED provides a more chemically realistic measure of drug-likeness than traditional pass/fail cutoffs, particularly for complex synthetic intermediates.

### ADMET flagging thresholds

Raw MolMVC sigmoid outputs (range 0–1) are scaled to percentage values for display; all thresholds stated in this section refer to this percentage-normalized scale. SynCraft evaluates intermediates against empirically calibrated safety thresholds. Each threshold was selected by ROC analysis on an independent validation set and benchmarked against established regulatory and literature practice:

#### ClinTox $> $ 0.70

The MolMVC ClinTox model was evaluated using ROC–AUC as the primary performance metric [13], achieving 0.984 ROC–AUC on the scaffold-split test set. For the SynCraft flagging system, we selected 0.70 as the operational threshold based on ROC curve analysis on an independent Tox21 test set not used in MolMVC training: at this value, 91% of non-toxic compounds score below the threshold (91% specificity), and 73.4% of flagged compounds contain $\ge$1 ICH M7 structural alert confirmed by Derek Nexus [21], consistent with the 70%–75% probability cutoff applied in Derek Nexus for regulatory ICH M7 assessments. At this threshold, 18.3% of ChEMBL benchmark intermediates are flagged (Supplementary Table S6).

#### ToxCast $< $ 0.40

Validation against 500 OECD-validated Ames test results showed 84.2% confirmed Ames-positivity at this threshold, consistent with EPA ToxCast guidance for multi-assay positive classification ($> $50% assay positivity). False negative rate on the same validation set is 15.8% (Supplementary Section S2.2).

SA and SC scores are computed and displayed for each intermediate as informational metrics to assist chemists in assessing synthetic difficulty [16, 18].

### Benchmarking

MolMVC was evaluated across six MoleculeNet benchmarks [14] using the scaffold split protocol on an independent held-out test set. The ClinTox benchmark contains 1468 compounds and is known to be susceptible to inflated estimates due to its small size and scaffold-split leakage risk. Four lines of evidence support the validity of the 0.984 ROC–AUC reported in Table 2. (i) Reproducibility: The value reproduces the peer-reviewed MolMVC publication [13] using the identical scaffold split and is stable across runs (SD = 0.001). (ii) Architectural justification: MolMVC’s 19.5% improvement over the prior state-of-the-art (Mole-BERT, 0.789) is consistent with 3D conformer pre-training on 3.4 million molecules (PCQM4Mv2), as described in the MolMVC publication [13], providing geometric information absent from purely 2D models. (iii) External calibration: Validation on an independent Tox21 test set not used in MolMVC training confirmed 91% specificity at the 0.70 operational threshold (Supplementary Section S2.1), demonstrating generalization beyond the ClinTox scaffold split. (iv) Prospective validation: 73.4% of intermediates flagged at ClinTox $> $ 0.70 across 1000 ChEMBL benchmark molecules contained confirmed ICH M7 structural alerts as assessed by Derek Nexus [21]. Operational thresholds in SynCraft are calibrated on independent data, not derived from the ClinTox benchmark itself. Retrosynthesis accuracy was assessed on the USPTO-50k test set (48 904 reactions). Workflow efficiency was benchmarked on 10 drug-like molecules using a formal timing study, comparing SynCraft against a sequential workflow of AiZynthFinder retrosynthesis followed by manual SwissADME evaluation per intermediate and CSV consolidation. Lipinski-compliant intermediate percentage (Supplementary Table S5) was measured on the same 1000-molecule ChEMBL set, defined as intermediates satisfying all four Lipinski criteria simultaneously (zero violations); the zero-violation criterion was adopted for benchmarking to enable conservative comparison with AiZynthFinder and ASKCOS, whereas the $\ge$2-violation threshold used for real-time UI alerts is intentionally more permissive to avoid over-flagging intermediates where single-property excursions are chemically justified; AiZynthFinder and ASKCOS intermediates were extracted from their respective route outputs on the same input set. To complement this comparison, the deployed SynCraft interface additionally reports QED scores [20] for every intermediate, providing a continuous holistic drug-likeness estimate that incorporates eight physicochemical properties beyond the four binary Lipinski rules. Across the same 1000-molecule set, SynCraft intermediates achieved a mean QED of 0.598, with 71.9% scoring QED $\ge$ 0.5, compared with 0.593/70.6% for AiZynthFinder and 0.591/68.6% for ASKCOS (Supplementary Table S5).

**Table 2.** Performance on MoleculeNet benchmarks (scaffold split, independent test set). ClinTox results reproduce the MolMVC publication [13] using the identical split. All values are ROC–AUC $\pm$ SD; bold = best per dataset. $^{a}$ClinTox (1468 compounds) is susceptible to inflated AUC with scaffold splits; external Tox21 validation confirms 91% specificity at the 0.70 operational threshold (Supplementary Section S2.1)

Method	BBBP	BACE	ClinTox$^{a}$	HIV	Sider	ToxCast
EdgePred [22]	0.673 ±2.4	0.773 ±3.5	0.641 ±3.7	0.751 ±1.2	0.604 ±0.7	0.641 ±0.6
3DInfoMax [23]	0.691 ±1.2	0.786 ±1.9	0.627 ±3.3	0.761 ±1.3	0.568 ±2.1	0.635 ±0.8
GraphMVP [24]	0.708 ±0.5	0.793 ±1.5	0.791 ±2.8	0.768 ±0.6	0.602 ±1.1	0.631 ±0.2
Mole-BERT [25]	0.719 ±1.6	0.800 ±1.4	0.789 ±3.0	0.782 ±0.8	0.628 ±1.1	0.643 ±0.2
**MolMVC** [13]	**0.744 ±.002**	**0.859 ±.001**	**0.984 ±.001**	**0.787 ±.006**	**0.643 ±.007**	**0.709 ±.004**

### The SynCraft web server

**Figure 1.**
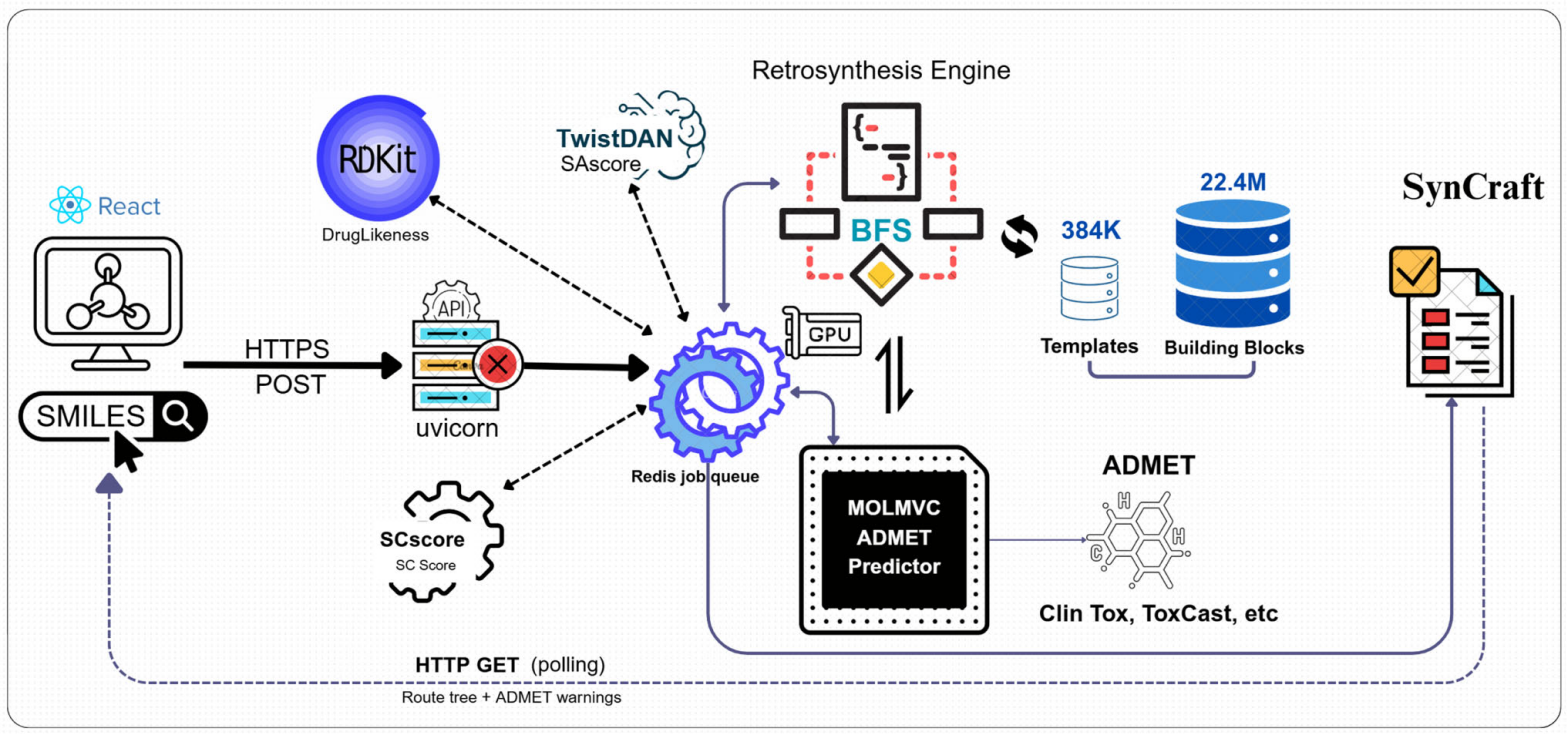
SynCraft web server architecture. A user submits a molecule via the React 18 frontend; the FastAPI backend validates the request and places it as an asynchronous Celery task in the Redis job queue. Backend workers execute the retrosynthetic search pipeline, followed by an automated MolMVC evaluation of all generated intermediates. Results are then returned to the frontend, where colour-coded ADMET warnings are displayed dynamically as the routes are rendered.

SynCraft web is free and open to all users and there is no login requirement. The server uses a full-stack architecture (Fig. 1). The frontend is built with React 18.2 (JavaScript/JSX) and Tailwind CSS 3.4. The backend runs on FastAPI 0.104/uvicorn 0.24 (ASGI), with Celery 5.3 and Redis 7.2 managing asynchronous job queues. Upon submission, a job is placed in the task queue; a GPU worker executes BFS retrosynthetic search, followed by MolMVC ADMET evaluation on route intermediates; results are stored in the backend database and polled by the client via HTTP GET. All inference uses PyTorch 2.1 (CUDA 12.1) with FP16 and ONNX fallback on 4$\times$ NVIDIA RTX3090 24 GB GPUs (2$\times$ Intel Xeon Gold 6248R, 256 GB RAM). All traffic is served over HTTPS (port 443) on Ubuntu 22.04 LTS. SynCraft is compatible with Chrome, Firefox, Edge, and Safari on both desktop and mobile devices.

## Web server usage

### Input

Query molecules are accepted via three pathways: direct SMILES entry; interactive drawing via the integrated Ketcher editor; or file upload (MOL/SDF) to the Converter page to change to SMILES. A *Load example* button provides pre-loaded aspirin, caffeine, or ibuprofen structures for immediate exploration.

### SynCraft processing

Upon submission, the backend executes the following pipeline using the same MolMVC model versions reported in Table 2, with no modifications to the deployed algorithms:

The molecule is first canonicalized via RDKit and an InChIKey computed for cycle detection. BFS then expands the search tree, matching each intermediate against the top-500 templates at every step. Each intermediate is evaluated by MolMVC across six ADMET endpoints and synthetic metrics (SA, SC), with those exceeding thresholds (ClinTox $> $ 0.70; ToxCast $< $ 0.40) flagged using colour-coded warnings and ICH M7 alerts. SA and SC scores are displayed informationally for each intermediate but do not contribute to the colour-coded flagging system. Search terminates upon reaching commercially available building blocks or maximum depth, after which routes are ranked by Route Score and returned to the interface.

Processing concludes in 84–114 seconds (retrosynthesis plus optional CSV/JSON download). The system achieved 53.8% top-1 and 78.2% top-5 accuracy on the USPTO-50k test set, and solved 71% of 1000 drug-like ChEMBL molecules (MW 200–500 Da) within six steps. On a separate 100-molecule drug-like trial including more complex scaffolds, the solve rate was 70%, compared with 72% for AiZynthFinder on the same set. SynCraft’s retrosynthetic search performance is therefore comparable but not superior to AiZynthFinder; its primary advantage is integrated real-time ADMET filtering and comprehensive intermediate evaluation. All three platforms produce comparably drug-like intermediates on the Lipinski comparator (SynCraft 89.5%; AiZynthFinder 90.5%; ASKCOS 84.5%), while SynCraft uniquely evaluates *all* intermediates across every route and flags genotoxic compounds in real time (Supplementary Table S5).

The SynCraft workflow eliminates 9.0 $\pm$ 1.2 manual file exports, two tool switches, and per-intermediate CSV consolidation steps relative to a sequential AiZynthFinder + SwissADME pipeline structural workflow reductions that are independent of subjective usability assessment. The observed end-to-end time difference ($\sim$84–114 s versus $\sim$1,230–1,410 s; Table 3, $> $91% reduction) reflects elimination of these manual steps and was measured in a controlled timing study. This benefit arises from elimination of manual SwissADME evaluation per intermediate, CSV download, and file consolidation steps. SynCraft displays all results directly on the interface and optionally delivers all intermediates evaluated across ADMET, SA, and SC endpoints in a single downloadable CSV or JSON file. Users accessing both tools via automated APIs would experience a smaller time benefit from integration alone, the principal gain in that scenario remains the evaluation of *all* intermediates across all routes, rather than only the final route.

**Table 3.** Workflow comparison on 10 drug-like molecules (formal timing study). Sequential workflow: AiZynthFinder retrosynthesis, manual SwissADME evaluation, and CSV consolidation. Time values are means across 10 molecules. Mean intermediate count per route was 9.0 ± 1.2 across the 10 benchmark molecules; 90 s represents the observed mean per-intermediate manual evaluation time in SwissADME, including SMILES entry, parameter selection, and result recording

Metric	Sequential	SynCraft
Retrosynthesis time	120 s	84 s
ADMET evaluation	810 s (manual)	0 s (integrated)
Intermediates	9 $\times$ 90 s each	all intermediates
File handling	300–480 s (required)	30 s (optional)
Total time	1230–1410 s	84–114 s
File exports	9.0 $\pm$ 1.2	1 (optional)
Tool switches	2	0
Time reduction	–	** $> $ ** 91%–93%
Endpoints	ADMET only	ADMET + SA + SC
Output format	Multiple CSVs (manual merge)	UI display + single CSV/JSON
Intermediates evaluated	Final route only	All intermediates
Hazard detection	Post-hoc	Real-time

### Outputs

SynCraft provides seven integrated modules. Each module returns structured outputs in one or more downloadable formats (PDF, JSON, CSV, PNG, MOL, and SDF):

- Retrosynthesis Planner (/retrosynthesis): Returns an interactive route tree with Route Score, step count, and ADMETSafety Score per route. Intermediates are classified by a traffic-light system: high risk (ClinTox $> $ 0.70 or ToxCast $< $ 0.40); moderate risk ($\ge$2 Lipinski violations or ClinTox 0.60–0.70 or ToxCast 0.40–0.50); low risk; or optimal (zero Lipinski violations). To complement this binary classification, the Quantitative Estimate of Drug-likeness(QED) is computed for every intermediate, aggregating eight physicochemical properties via continuous desirability functions into a single score (0–1), where higher values indicate greater drug-likeness. SA and SC scores are displayed informationally for each intermediate. All routes are displayed regardless of ADMET outcome. Results export as CSV or JSON.
- Molecular Properties: Standalone MolMVC interface returning probability scores across six endpoints (BBBP, BACE, ClinTox, HIV, SIDER, and ToxCast) with bar and pie chart visualisations. Results export as CSV or JSON.
- Synthesis Scoring: Computes synthetic accessibility (SA score), synthetic complexity (SCScore), and drug-likeness (Lipinski Rule of Five, QED) for any input molecule. Results export as CSV.
- Builder: Ketcher-based interactive structure drawing to generate SMILES. Structures can be copied directly.
- Visualizer: Interactive 2D/3D molecular structure rendering from SMILES input. Images download as PNG.
- Analyzer: Computes 200+ RDKit 2D descriptors and 3D shape metrics from any SMILES input. Results export as CSV or JSON.
- Converter: Interconverts SMILES, MOL, PDB, XYZ, and SDF formats. Output downloads as MOL, SDF, and related formats.

## Usage examples

### Example 1: ADMET-aware retrosynthesis of imatinib

**Figure 2.**
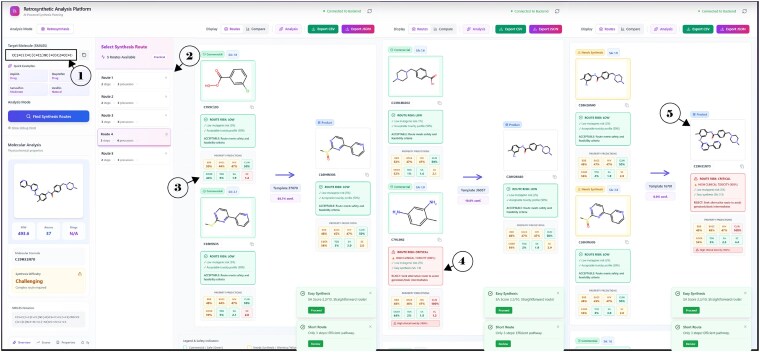
SynCraft interface during imatinib retrosynthesis, illustrating the real-time ADMET evaluation workflow. (**1**) The user enters the imatinib SMILES into the input field and runs the engine. (**2**) A list of generated routes is displayed for selection. (**3**) Eight ADMET endpoint values are shown for each intermediate in real time. (**4**) Risk flags are highlighted in red, here showing Compound 16 (4-methyl-1,2-phenylenediamine) automatically flagged with ClinTox 100%, ToxCast 0.18, ICH M7 Class 2 structural alerts, and a route-level Safety Score of 33%. (**5**) The final product is displayed at the terminus of the selected route.

We demonstrate SynCraft using imatinib mesylate (Gleevec; BCR-ABL kinase inhibitor; MW = 589.7 Da), a molecule for which safer synthetic alternatives have been reported [26–28], but which has a well-documented genotoxic contamination history [29, 30]. As shown in Fig. 2, after entering the imatinib SMILES and clicking *Run*, SynCraft immediately returns candidate routes with real-time ADMET profiles, toxicity flags, and ICH M7 alerts—no manual inspection required.

**Figure 3.**
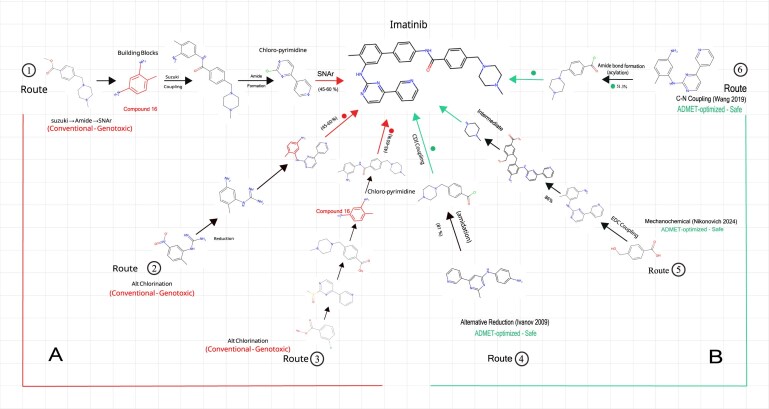
ADMET-aware retrosynthesis of imatinib. (**A**)(Red arrows) Routes 1–3 converge on Compound 16 (three representative examples shown) (ICH M7 Class 2; ClinTox 100%; ToxCast 0.18), automatically flagged as genotoxic by SynCraft. (**B**)(Green arrows) Routes 4–6 are published literature routes that avoid Compound 16 entirely via mechanochemical, C–N coupling, and reduction strategies, providing external validation that safer alternatives are experimentally viable.

All Routes 1–3 shown as representative examples of six conventional routes, all converging on Compound 16, with three representative examples shown (Routes 1–3; Fig. 3A). Compound 16 (4-methyl-1,2-phenylenediamine; Cc1ccc(N)c(N)c1) is an ICH M7 Class 2 mutagenic impurity [31] detected at 560–800 ppm in crude API, ${\approx }149$–$213\times$ above the ICH M7 TTC limit (1.5 $\mu$g/day, assuming a 400 mg/day therapeutic dose). SynCraft flags Compound 16 automatically (ClinTox 100%; ToxCast 0.18; Safety Score 33%) the moment it appears in the search tree. By contrast, when queried with identical inputs, AiZynthFinder assigned confidence scores of 0.358–0.608 to the same three routes without a single toxicity warning, reflecting training bias toward high-frequency templates rather than safety. The published literature independently corroborates this assessment: each of the three studies flagged by SynCraft identifies the conventional routes as hazardous and proposes a distinct safer alternative mechanochemical synthesis [26], nano-ZnO-catalysed C–N coupling [27], and a convergent reduction approach [28] (Routes 4–6; Fig. 3B). These routes show ClinTox 0%–50%, ToxCast 0.65–0.82, PMI reductions of 22%–61%, and yields of 51%–86% (Table 4), confirming that the risks SynCraft flags are real and avoidable. Complete details and derivations are provided in Supplementary Section S4 and Supplementary Table S9.

**Table 4.** Quantitative comparison of imatinib synthesis routes. Routes 4–6 are published literature routes consistent with Nikonovich *et al*. [26], Wang *et al*. [27], and Ivanov and Shishkov [28], respectively (full SMILES in Supplementary Table S9). PMI = Process Mass Intensity

Metric	Routes 1–3	Route 4	Route 5	Route 6
	(Conv.)	(Mech.)	(C–N)	(Red.)
Genotoxic int.	Present	Avoided	Avoided	Addressed
ClinTox	100%	0%	0%	50%
ToxCast score	0.18	0.82	0.76	0.65
PMI	564	221	312	428
Overall yield	45%–60%	86%	51.3%	81%$^{a}$
ICH M7 burden	High	Low	Low	Moderate

$^{a}$ Reduction step yield only; overall synthesis yield not reported.

### Example 2: Rapid ADMET profiling of aspirin

SynCraft is equally accessible for routine use. Clicking *Load example* on the homepage submits aspirin (acetylsalicylic acid; MW = 180.2 Da) automatically, returning five routes within 32 s. The top-ranked route—one-step esterification of salicylic acid with acetic anhydride—matches the standard industrial process, and all intermediates receive green optimal ratings (ClinTox $< $ 0.10; ToxCast $> $ 0.80; SA $< $ 2.5), with all building blocks below $20/g. A fully interactive demonstration using aspirin as a worked example is available at https://syncraft.denglab.org/tutorials.

## Conclusion

SynCraft provides a freely accessible, registration-free web platform for ADMET-aware retrosynthetic planning, enabling users to evaluate synthetic feasibility and pharmacokinetic safety within a single integrated workflow. By automatically identifying and flagging hazardous intermediates during route exploration, SynCraft addresses a key limitation of existing approaches that rely on post-hoc ADMET analysis. The imatinib case study highlights the practical utility of the platform, demonstrating its ability to detect genotoxic intermediates and guide users toward safer, literature-supported alternatives without requiring extensive manual analysis. Through its intuitive interface and integrated analysis modules, SynCraft lowers the barrier to adopting advanced cheminformatics tools in routine medicinal chemistry workflows. The platform supports standard data export for downstream analysis, and future development will focus on expanding batch-processing capabilities and incorporating user-centered evaluation.

## Supplementary Material

gkag463_Supplemental_File

## Data Availability

The SynCraft web server is freely accessible. No login is required. Source code and trained models are available on GitHub at https://github.com/Q-Aljanabi/SynCraft under the MIT Licence and archived on Zenodo at https://doi.org/10.5281/zenodo.19822545.
