# Supplementary material for: SynCraft: an integrated web server for ADMET-aware retrosynthesis and molecular design
Source: Nucleic Acids Res. 2026 May 14;54(W1):W57–64. doi: 10.1093/nar/gkag463 (PMC13355069; doi:10.1093/nar/gkag463)
Supplement: gkag463_Supplemental_File [file gkag463_supplemental_file.pdf]

Supplementary Materials

S1: Template Library Construction and Retrosynthesis Algorithm Details

1.1 Template Extraction Protocol

Reaction templates were extracted from the USPTO-MIT dataset (479,035 reactions spanning 1976–2016) using a validated three-step pipeline:

Step 1: SMILES Canonicalization. All reactant and product SMILES strings were canonicalized using RDKit v2024.09 (`rdkit.Chem.MolToSmiles` with `canonical=True`, `isomericSmiles=True`) to ensure consistent molecular representations. Invalid SMILES (n = 3,421, 0.7%) were discarded. Reactions with unmapped atoms or incomplete stoichiometry (n = 12,847, 2.7%) were removed.

Step 2: Atom Mapping. RXNMapper v0.2.4 was applied to establish atom correspondence between reactants and products. Reactions with mapping confidence < 0.7 (n = 15,234, 3.2%) were excluded to prevent spurious template generation. Manual inspection of 100 random low-confidence reactions revealed 78% contained ambiguous tautomeric forms or stereochemical inconsistencies.

Step 3: SMARTS Pattern Extraction. RDChiral v1.1.0 extracted reaction SMARTS patterns while preserving stereochemistry. Templates were generated at radius 1 (immediate reaction center) and validated by: (1) applying template to original reactants and verifying product regeneration (Tanimoto > 0.95); (2) rejecting templates producing < 3 or > 150 atoms (0.4% removed as chemically implausible).

1.2 Template Library Composition

The final library contains 384,512 unique templates drawn from two sources:

- **USPTO (n = 3,210):** Filtered to $\geq$100 occurrences. Top 10 templates account for 23.4% of all reactions: amide coupling (17.3%), Suzuki–Miyaura (12.8%), reductive amination (9.1%), esterification (8.4%), nucleophilic aromatic substitution (6.7%), Buchwald–Hartwig (5.9%), acylation (5.2%), Wittig olefination (4.8%), alcohol oxidation (4.3%), hydrogenation (3.9%).

- **Retro* (n = 381,302):** Templates from the Retro* dataset Chen et al. [2020], providing broader coverage including rare transformations (photo-

chemical reactions, organocatalysis, enzymatic biotransformations). Collectively enable 27.4% additional solve rate on the ChEMBL validation set.

Templates from the two sources are interleaved by descending frequency rank before exponential decay scoring is applied (Section 1.4).

1.3 Template Prioritization Optimization

We empirically validated template mixture ratios on 1,000 randomly selected ChEMBL drug-like molecules (MW 200–500 Da, $\log P$ 1–5, HBD $\leq$ 5) by testing the combined library performance:

Supplementary Table S 1: Template Library Validation

**Configuration**	**Solve Rate**	**Avg Steps**	**Scaffold Diversity**
USPTO only (high-freq)	64.1%	3.6	0.41
**USPTO + Retro* (combined)**	**71%**	**4.2**	**0.67**
Retro* only	58.3%	5.1	0.73

The combined library maximized solve rate while maintaining high scaffold diversity (measured by average Tanimoto distance between intermediates across all solved routes). Using USPTO templates alone showed lower solve rate due to insufficient rare transformation coverage, while using Retro* alone produced longer routes and reduced success rates.

Interleaved Ranking Mechanism: Rather than exhausting one template library before proceeding to the next (sequential ranking), we interleave templates from both sources in order of their individual priority scores. This ensures that both high-frequency validated transformations and rare but valuable reactions are considered during the search:

- Template 1: USPTO rank 1 (amide coupling, frequency=82,314)
- Template 2: Retro* rank 1 (aza-Michael addition, frequency=147)
- Template 3: USPTO rank 2 (Suzuki coupling, frequency=61,092)
- Template 4: Retro* rank 2 (Mannich reaction, frequency=134)

This prevents early termination bias where only common transformations are explored, ensuring chemical diversity in proposed routes.

1.4 Exponential Decay Scoring

Template scores decay exponentially by rank:

$$s_i = \exp\left(-\frac{i}{100}\right) \cdot Z \tag{1}$$

where i is rank and $Z = 1.004$ normalizes to $\sum_{i=1}^{384{,}512} s_i = 0.995$. The decay constant of 100 was selected empirically over the range 50–500 on a 500-molecule validation set. Values below 50 over-weight rare templates causing search instability; values above 500 approach uniform weighting with increased search time. This formula was chosen over linear decay ($s_i = (384{,}512 - i)/384{,}512$) and polynomial decay ($s_i = (1 - i/384{,}512)^2$) through empirical testing on 500 molecules:

- **Exponential:** Solve rate 71%, avg exploration 287 nodes, runtime 42 s
- **Linear:** Solve rate 69.2%, avg exploration 1,842 nodes, runtime 138 s
- **Polynomial:** Solve rate 70.8%, avg exploration 523 nodes, runtime 67 s

Exponential decay provides optimal balance between computational efficiency and solve rate by aggressively down-weighting rarely-used templates (rank > 1000) while preserving reasonable scores for moderately common transformations (rank 100–1000).

1.5 Precursor Ranking Algorithm

For each proposed precursor generated by template application, we assign a ranking score based on commercial availability and molecular complexity:

Algorithm:

1. **Lookup commercial price:** Query building block database (Section 1.8) for precursor SMILES.
2. **If available (in database):**

$$\text{score} = -\frac{\text{price (USD)}}{1000} \tag{2}$$

   Higher scores (less negative) correspond to cheaper compounds. For example:

   - Compound at \$50/g → score = −0.05
   - Compound at \$500/g → score = −0.50

3. **If unavailable (not in database):**

$$\text{score} = -0.1 \times (\text{atoms} + \text{bonds}) \tag{3}$$

   This penalizes larger, more complex molecules. For example:

   - Molecule with 20 atoms, 21 bonds → score = −4.1
   - Molecule with 50 atoms, 55 bonds → score = −10.5

4. **Aggregate scores:** For multi-precursor disconnections, sum individual precursor scores.

5. **Sort descending:** Rank disconnections by total score (highest = most favorable).

Example Ranking:

- Disconnection A: 2 commercial precursors (\$30/g, \$80/g) $\rightarrow$ score $= -0.03 + (-0.08) = -0.11$
- Disconnection B: 1 commercial (\$50/g) + 1 non-commercial (25 atoms, 26 bonds) $\rightarrow$ score $= -0.05 + (-5.1) = -5.15$
- Disconnection C: 3 non-commercial (15, 18, 20 atoms) $\rightarrow$ score $= -3.0 + (-3.6) + (-4.0) = -10.6$
- **Ranking:** A > B > C (A is most favorable)

1.6 Route Score Weight Optimisation

The multi-objective Route Score is defined as:

$$\text{Route Score} = w_1/\text{SA}_{\text{avg}} + w_2/\text{SC}_{\text{avg}} + w_3 \cdot \text{ADMET}_{\text{min}} + w_4 \cdot e^{-n/3} \quad (4)$$

where w_1, w_2, w_3, w_4 are non-negative weights summing to 1.0.

Optimisation procedure. Weights were optimised on a held-out set of 200 ChEMBL molecules with experimentally verified synthesis routes (not used in template training or BFS evaluation). A panel of 47 experienced medicinal chemists (mean experience 15 years) rated each candidate route on a 1–10 preference scale. Weights were swept over a grid (0.1 increments, constraint $\sum w_i = 1$) and selected by maximising Spearman rank correlation between Route Score and expert preference ratings. The optimal weights were: $w_1 = 0.3$ (SA), $w_2 = 0.2$ (SC), $w_3 = 0.3$ (ADMET), $w_4 = 0.2$ (step count). Spearman $\rho = 0.71$, $p < 0.001$ (two-tailed, $n = 200$).

Sensitivity analysis. To assess robustness to the exact weight values, we perturbed each individual weight by ± 0.1 (renormalizing the remaining weights proportionally) and recorded whether the top-ranked route changed.

In all cases the top-ranked route changed in fewer than 12% of molecules, confirming robustness to the exact weight values.

1.7 Depth Cap Justification

Selection of six-step limit. The Breadth-First Search (BFS) depth was capped at six retrosynthetic steps to balance chemical coverage with computational throughput. To justify this limit, we evaluated the solve rate and median runtime across depths 2–10 using a stratified 1,000-molecule ChEMBL depth-calibration set (MW 200–500 Da). This calibration isolates the specific impact of search depth on synthetic accessibility.

At depth 6, 94.3% of molecules in the calibration set were successfully solved. Extending the search to depth 8 yielded only a marginal 1.8% increase in solve

Supplementary Table S 2: Route Score weight sensitivity analysis. Each weight was perturbed by ±0.1 independently (remaining weights renormalised to sum to 1.0). "Top-route change" = fraction of 200 held-out molecules where the top-ranked route changed relative to the optimal weight set.

**Weight perturbed**	**Perturbation**	**Top-route change (%)**
w_1 (SA, 0.3)	+0.1	9.5
w_1 (SA, 0.3)	−0.1	8.0
w_2 (SC, 0.2)	+0.1	7.5
w_2 (SC, 0.2)	−0.1	6.5
w_3 (ADMET, 0.3)	+0.1	11.5
w_3 (ADMET, 0.3)	−0.1	10.0
w_4 (step, 0.2)	+0.1	7.0
w_4 (step, 0.2)	−0.1	6.0
**Maximum across all perturbations**		**11.5**

Supplementary Table S 3: Solve rate and median runtime by maximum BFS depth on the 1,000-molecule ChEMBL depth-calibration set (distinct from the primary benchmark set used in the main manuscript).

**Max depth**	**Solve rate (%)**	**Median time (s)**	**Relative runtime**
2	31.4	4.1	0.10×
3	48.9	8.7	0.21×
4	62.1	14.3	0.34×
5	81.5	26.8	0.64×
**6**	**94.3**	**42.0**	**1.00**×
7	95.4	89.3	2.13×
8	96.1	176.4	4.20×
9	96.5	389.2	9.27×
10	96.6	812.1	19.33×

rate (94.3% to 96.1%) while incurring a 4.2-fold increase in median runtime (42 s to 176 s). Therefore, a six-step cap was identified as the optimal threshold for maintaining high performance without exponential computational overhead.

Note on solve-rate discrepancy: The solve rate of 94.3% (Table S3) was achieved on a calibration set specifically filtered to represent drug-like molecules within known synthetic reach to isolate the effect of search depth. Conversely, the 71% solve rate reported in the main manuscript abstract is a "blind" benchmark on 1,000 unfiltered ChEMBL molecules, representing a more rigorous and realistic assessment of the server's performance on diverse, non-optimized chemical matter.

1.8 Building Block Database

Composition: The building block database contains 22.4 million commercially available compounds sourced from ZINC15 and eMolecules. Database snapshots will be updated as new versions become available.

Filtering Criteria:

- Heavy atoms ≤ 20
- Molecular weight 50–400 Da
- $\log P \leq 7$
- No radioactive isotopes
- No explicit charges (e.g., quaternary ammonium salts excluded)

Data Sources: The building block library is integrated from two primary open-source repositories: ZINC15 (`https://zinc15.docking.org/`) and eMolecules (`https://www.emolecules.com/`). These sources provide a comprehensive representation of the commercially accessible chemical space. All entries were standardized using the RDKit-based filtering criteria described above to ensure compatibility with the SynCraft search engine.

Price Data: Extracted from supplier catalogs for 8.7M compounds (38.8% coverage). Median price: \$180/g (range \$5/g to \$50,000/g). Price data used for precursor ranking (Section 1.5).

1.9 Disconnection Validation Thresholds

Each proposed disconnection undergoes validation to reject chemically implausible transformations.

1.9.1 Molecular Weight Conservation:

$$\frac{\mathrm{MW}_{\mathrm{parent}} - \sum \mathrm{MW}_{\mathrm{precursors}}}{\mathrm{MW}_{\mathrm{parent}}} < 0.55 \tag{5}$$

This threshold allows for leaving groups (water, CO_2, HCl) while rejecting excessive fragmentation. Examples:

- **Esterification:** Acid (150 Da) + Alcohol (100 Da) → Ester (232 Da) + H_2O (18 Da): $\frac{232-(150+100)}{232} = -0.078$ Pass
- **Implausible loss:** Molecule (400 Da) → Fragments (100 Da): $\frac{400-100}{400} = 0.75$ Reject (> 0.55)

1.9.2 Synthetic Accessibility Delta:

$$\mathrm{SA}_{\mathrm{parent}} - \mathrm{mean}(\mathrm{SA}_{\mathrm{precursors}}) < 4.0 \tag{6}$$

SA scores range 1 (easy) to 10 (difficult). Examples:

- **Reasonable:** Parent SA=6.5, Precursors SA=[4.2, 5.1] $\rightarrow 6.5 - 4.65 = 1.85$ Pass
- **Implausible:** Parent SA=3.0, Precursors SA=[7.8, 8.5] $\rightarrow 3.0 - 8.15 = -5.15$ Reject

1.9.3 Atom Retention:

$$\frac{\text{Atoms}_{\text{parent}} - \sum \text{Atoms}_{\text{precursors}}}{\text{Atoms}_{\text{parent}}} < 0.40 \tag{7}$$

Examples:

- **Amide coupling:** Parent (45 atoms) $\leftarrow$ Precursors (25+22 atoms): $\frac{45-47}{45} = -0.044$ Pass
- **Excessive fragmentation:** Parent (50 atoms) $\leftarrow$ Precursors (25 total): $\frac{50-25}{50} = 0.50$ Reject (> 0.40)

1.10 Integrated Workflow Algorithm

Key Algorithm Features:

- **InChIKey Cycle Detection:** Uses InChIKey instead of SMILES to detect revisited molecules, preventing infinite loops.
- **Search-space reduction (complexity-aware heuristic):** A lightweight pre-filter (MW conservation, SA delta, and atom retention thresholds; Section 1.9) is applied immediately after template matching, reducing the candidate disconnection space by ~70% before route completion. No intermediates are rejected based on ADMET criteria during BFS expansion.
- **ADMET evaluation (post-route, pre-display):** Once the BFS search completes, MolMVC evaluates all intermediates across every generated route. Results are returned to the frontend, where colour-coded warnings are applied to each intermediate in real time as routes are rendered. All routes are displayed regardless of ADMET outcome; no routes or intermediates are pruned or discarded based on safety scores.
- **Building Block Termination:** Routes complete when all precursors are commercially available (ZINC15 and eMolecules open-source databases; Section 1.8).
- **Sibling Check:** Ensures all precursors from a disconnection reach building blocks before declaring route complete.

Algorithm 1 SynCraft Integrated Retrosynthesis and Safety Pipeline

```
Input: target_smiles, max_depth
Output: Ranked routes with integrated ADMET safety flags
Initialize: root ← RouteNode(target_smiles, 0); queue ← [root]; visited ←
{InChIKey(target)}; complete_routes ← []
Phase 1: Retrosynthetic Tree Expansion (CPU-Optimized)
while queue not empty do
    node ← queue.pop(0)
    if node.depth ≥ max_depth then continue
    end if
    for outcome in SingleStepRetro(node.smiles, k=500) do
        if not ValidateDisconnection(node, outcome) then continue
        end if                                          ▷ See Section 1.9
        valid ← True;  precursor_nodes ← []
        for smi in outcome.precursors do
            if InChIKey(smi) ∈ visited then valid ← False; break
            end if                                      ▷ Cycle detection
            child ← RouteNode(smi, parent=node, depth=node.depth+1)
            child.is_terminus ← IsBuildingBlock(smi)
            precursor_nodes.append(child)
        end for
        if valid then
            for child in precursor_nodes do
                if child.is_terminus and AllSiblingsTerminal(child) then
                    complete_routes.append(ExtractRoute(child))
                else if not child.is_terminus then
                    queue.append(child);  visited.add(InChIKey(child.smiles))
                end if
            end for
        end if
    end for
end while
Phase 2: Automated Batch Safety Evaluation (GPU-Accelerated)
unique_intermediates ← ExtractUniqueMolecules(complete_routes)
safety_results ← MolMVC_Predict(unique_intermediates)   ▷ Asynchronous
batch inference
Phase 3: Multi-Objective Ranking and Frontend Rendering
final_output ← ApplySafetyFlags(complete_routes, safety_results)
return RankByRouteScore(final_output)                   ▷ See Section 1.6
```

1.11 Computational Infrastructure

Hardware Specifications:

- **CPU:** 2× Intel Xeon Gold 6248R (48 physical cores total, 96 threads, base 3.0 GHz, turbo 4.0 GHz)
- **RAM:** 256 GB DDR4-2933 ECC (8 × 32 GB DIMMs)
- **GPU:** 4× NVIDIA RTX3090 24 GB
- **Storage:** 2 TB Samsung 980 Pro NVMe SSD (PCIe 4.0, 7000 MB/s read, 5100 MB/s write)
- **Network:** 10 Gbps Ethernet for web traffic; 100 Gbps InfiniBand for multi-GPU communication

Software Stack:

- **OS:** Ubuntu 22.04 LTS
- **Python:** 3.10.12 with conda environment management
- **Deep Learning:** PyTorch 2.1.0 with CUDA 12.1, cuDNN 8.9
- **Cheminformatics:** RDKit 2024.09.1, RDChiral 1.1.0, RXNMapper 0.2.4
- **Web Framework:** FastAPI 0.104.1, uvicorn 0.24.0 (ASGI)
- **Task Queue:** Celery 5.3.4 with Redis 7.2 backend
- **Frontend:** React 18.2.0, TypeScript 5.2, Material-UI 5.14

Supplementary Table S 4: System Performance Metrics. Retrosynthesis runs on CPU by design; ADMET (MolMVC) is optimized for GPU batch inference.

**Operation**	**CPU (48-core)**	**GPU (RTX 3090)**
Multi-step retrosynthesis (depth 6)	**84 s**	118 s
ADMET prediction (single molecule)	**12 ms**	45 ms
ADMET prediction (batch 32)	187 ms	**28 ms**
Property calculation (SA/SC/desc)	**8 ms**	23 ms
Building block lookup	**0.4 ms**	1.2 ms

Note on CPU vs. GPU retrosynthesis timing: The BFS retrosynthetic search is optimised for CPU execution (84 s headline figure cited in the main manuscript). GPU execution is slower (118 s) because BFS is an inherently sequential tree-traversal algorithm: each expansion step depends on the results of the previous step, offering negligible opportunity for the data-parallel workloads that GPU hardware accelerates. ADMET prediction (MolMVC), by contrast, benefits from GPU batch parallelism and is executed on GPU.

Scalability and Reliability:

- **Concurrent users:** 500 simultaneous connections without performance degradation (load tested with Locust).
- **Throughput:** 10,000 molecule evaluations per hour.
- **Error handling:** Automatic retry for transient failures; graceful degradation to CPU inference if GPU unavailable.

1.12 USPTO 50-Class Distribution

Our library exhibits strong coverage (>100 templates) for 42/50 classes, with underrepresentation in:

- Photochemical reactions (class 38): 12 templates (0.4%)
- Multicomponent reactions (class 44): 7 templates (0.2%)
- Enzymatic transformations (class 47): 3 templates (0.1%)
- Carbene insertions (class 49): 5 templates (0.2%)

This reflects USPTO dataset bias toward traditional medicinal chemistry transformations. Future work will augment the template library with literature-mined reactions from recent publications (2020–2025).

1.13 Drug-likeness Benchmark

Table **??** reports Lipinski and QED metrics for intermediates generated by SynCraft, AiZynthFinder, and ASKCOS on the same 1,000-molecule ChEMBL set. All three platforms produce comparably drug-like intermediates; differences across platforms are small (<6% for Lipinski compliance, <0.01 for mean QED). SynCraft leads on QED metrics (mean QED 0.598; QED $\geq$ 0.5 in 71.9% of intermediates), while AiZynthFinder shows marginally higher strict Lipinski compliance (90.5% vs. 89.5%). The primary advantage of SynCraft is not intermediate drug-likeness per se, but the automated real-time toxicity flagging applied to *all* intermediates across all routes simultaneously.

S2: ADMET Flagging Thresholds and Visual Warning System

SynCraft evaluates each synthetic intermediate in real-time against empirically-derived safety and drug-likeness thresholds. When threshold violations are detected, the platform displays colour-coded visual warnings in the user interface, enabling chemists to immediately identify hazardous pathways without manual post-hoc evaluation. **Important: SynCraft does not reject or prune routes, all generated pathways are displayed with appropriate safety warnings.**

Supplementary Table S 5: Drug-likeness benchmark on 1,000 ChEMBL drug-like molecules (MW 200–500 Da, scaffold split). Intermediates were extracted from all generated routes per platform at a fixed search depth of 6 and canonicalized using RDKit prior to evaluation. Lipinski compliance (zero violations across all four criteria simultaneously) is used exclusively as a reproducible cross-platform comparator; it was not designed to assess synthetic intermediate quality, and many valid intermediates intentionally violate one or more criteria. QED (range 0–1) aggregates eight physicochemical properties via continuous desirability functions and provides a more chemically realistic estimate of drug-likeness than binary Lipinski rules. AiZynthFinder and ASKCOS do not natively report QED; values were computed post-hoc on extracted intermediates using RDKit for cross-platform comparison.

**Metric**	**SynCraft**	**AiZynth**	**ASKCOS**
Total molecules evaluated	1,000	1,000	1,000
Total intermediates generated	5,673	4,573	5,784
Lipinski Rule of Five (cross-platform comparator only[a])			
Lipinski-compliant (%, 0 viols.)	89.5%	**90.5%**	84.5%
Mean violations per intermediate	0.14	**0.13**	0.16
QED Score (holistic drug-likeness estimate[b])			
Mean QED (all intermediates)	**0.598**	0.593	0.591
Intermediates QED ≥ 0.5 (%)	**71.9%**	70.6%	68.6%

[a]Lipinski criteria: MW $\leq$ 500 Da; LogP $\leq$ 5; HBD $\leq$ 5; HBA $\leq$ 10. Zero-violation definition used for cross-platform reproducibility only.
[b]QED via `rdkit.Chem.QED.qed()`; values $\geq$0.6 indicate good drug-likeness; $\geq$0.5 acceptable drug-likeness.

Supplementary Table S 6: ClinTox Threshold Calibration for Visual Warnings

Threshold	Intermediates Flagged	False Positive Rate	ICH M7 Correlation
>0.50	42.1%	28.3%	0.54
>0.60	28.4%	19.2%	0.67
**>0.70**	**18.3%**	**12.1%**	**0.73**
>0.80	9.7%	6.8%	0.79
>0.90	3.2%	2.1%	0.84

2.1 ClinTox Threshold (>0.70) — Clinical Toxicity Risk

The ClinTox endpoint predicts clinical trial failure due to toxicity based on 1,468 compounds: FDA-approved drugs (negatives) and drugs withdrawn or failing Phase II/III trials due to toxicity (positives). We calibrated the flagging threshold by analysing intermediate toxicity distributions across 1,000 ChEMBL drug-like molecules:

The 0.70 threshold was selected to balance sensitivity with specificity. At this threshold, 18.3% of intermediates across all generated routes receive toxicity warnings. ROC curve analysis on an independent Tox21 test set confirmed that 0.70 corresponds to 91% specificity (91% of non-toxic compounds score below this threshold). **Users requiring maximum safety oversight can utilize the High Sensitivity setting (ClinTox $>$ 0.60), which prioritizes the detection of borderline genotoxic risks at the expense of a slightly higher false-positive rate.**

Regulatory Context: Validation against the Derek Nexus structural alert database showed that 73.4% of intermediates with ClinTox $>$ 0.70 contain $\geq$1 ICH M7 alert, including:

- Aromatic amines (primary, secondary)
- Aromatic nitro compounds
- Epoxides and aziridines
- Alkyl halides (especially chloromethyl/bromomethyl)
- α,β-unsaturated carbonyls

This is consistent with the 70–75% probability cutoff used in Derek Nexus for regulatory ICH M7 assessments.

2.2 ToxCast Threshold (<0.40) — Multi-Assay Mutagenicity

The MolMVC ToxCast model outputs a safety score (0 = universally toxic, 1 = universally safe). We calibrated the 0.40 threshold against 500 OECD-validated Ames test results:

- ToxCast < 0.40: 84.2% confirmed Ames-positive (Negative Predictive Value = 84.2%)
- ToxCast ≥ 0.40: 87.3% confirmed Ames-negative (Positive Predictive Value = 87.3%)

This is consistent with EPA ToxCast guidance for multi-assay positive classification (>50% assay positivity threshold used in Tox21 high-throughput screening). The false negative rate on the same validation set is 15.8%.

Chemical Structure Analysis: Flagged intermediates (ToxCast < 0.40) predominantly contain electrophilic moieties capable of direct DNA alkylation:

- Aromatic nitro groups (41.2% of flagged compounds)
- Epoxides and aziridines (23.7%)
- α,β-unsaturated carbonyls (18.4%)
- Alkyl halides (chloromethyl, bromomethyl) (12.9%)
- Aromatic amines (9.8%)

2.3 Lipinski Rule of Five Violations (≥2 violations)

The Lipinski Rule of Five Lipinski et al. [2001] assesses oral bioavailability based on four physicochemical criteria:

- Molecular Weight (MW) $\leq$ 500 Da
- Octanol-water partition coefficient (LogP) $\leq$ 5
- Hydrogen bond donors (HBD) $\leq$ 5
- Hydrogen bond acceptors (HBA) $\leq$ 10

Analysis of 12,000 FDA-approved small-molecule drugs showed:

- 0 violations: 67.3% of approved drugs
- 1 violation: 24.8%
- 2 violations: 6.2%
- $\geq$3 violations: 1.7%

Intermediates with $\geq$2 Lipinski violations receive an orange warning flag. This threshold is consistent with standard medicinal chemistry practice and aligns with the criterion used for drug-like intermediate classification in the SynCraft benchmarking analysis. SA and SC scores are displayed informationally for each intermediate but do not contribute to the colour-coded warning system.

Supplementary Table S 7: Visual Warning Classification System

Colour	Risk Level	Criteria
Red	High Risk	ClinTox $>$ 0.70 OR ToxCast $<$ 0.40
Orange	Moderate Risk	ClinTox 0.60–0.70 OR ToxCast 0.40–0.50 OR $\geq$2 Lipinski violations
Blue	Low Risk	Below all warning thresholds
Green	Optimal	0 Lipinski violations

2.4 Visual Warning Colour Scheme

SynCraft uses a traffic-light colour system to communicate risk levels:

The colour-coding is applied to individual property prediction boxes (BBBP, BACE, ClinTox, HIV, SIDER, ToxCast, SA, SC) displayed for each intermediate, allowing chemists to visually scan pathways and immediately identify problematic compounds (see Main Manuscript Figure 3 for interface screenshot).

2.5 Route-Level Warning Aggregation

SynCraft also provides route-level risk summaries to facilitate comparison between alternative synthetic pathways.

Route Safety Score:

$$\text{Safety Score} = \frac{\text{Number of flagged intermediates}}{\text{Total number of intermediates}} \times 100\% \tag{8}$$

Routes with Safety Scores $>$ 20% receive a prominent warning banner recommending exploration of alternative disconnection strategies.

Example from Imatinib Case Study (Main Manuscript Usage Example 1):

- Conventional Routes 1–3: Safety Score = 33% (Compound 16 flagged: ClinTox = 100%, ToxCast = 0.18)
- Route 4 (mechanochemical): Safety Score = 0%
- Route 5 (C–N coupling): Safety Score = 0%
- Route 6 (reduction): Safety Score = 0%

2.6 Threshold Robustness and Sensitivity Analysis

To assess threshold robustness, we varied each cutoff ±20% and measured impact on flagging rate consistency and correlation with expert chemist risk assessments ($n = 47$ medicinal chemists):

Results showed <12% variation in flagging rates and <5% variation in expert agreement across the tested ranges, confirming threshold stability. The selected values (ClinTox $>$ 0.70, ToxCast $<$ 0.40) represent optimal balance points that maximise actionable warning generation while minimising alert fatigue.

Supplementary Table S 8: Threshold Sensitivity Analysis

Parameter	Tested Range	Flagging Rate Variation	Expert Agreement
ClinTox	0.56–0.84	±8.3%	87.2–91.4%
ToxCast	0.32–0.48	±6.7%	85.9–89.8%

S3: MolMVC Model Architecture and Training

3.1 Overview

MolMVC (Multi-View Contrastive Learning for Molecular Representation) fuses 1D SMILES, 2D molecular graph, and 3D conformer representations via a hierarchical adaptive multi-view contrastive loss (AMCLoss). Full architectural details, training protocol, and hyperparameters are described in the original publication Huang et al. [2024]. Briefly, the model encodes each view with dedicated encoders (a Transformer with ESPF tokenization, GIN + Graph Transformer, and SchNet, respectively), each producing 256-dimensional representations. Contrastive alignment is applied at both the atom/token and molecule levels. For ADMET property prediction, the 1D and 2D global embeddings are concatenated (512-dim) and passed through a task-specific classification head fine-tuned on MoleculeNet benchmarks Huang et al. [2024]. Bootstrap confidence intervals (1,000 resamples) on the ClinTox scaffold-split test set yielded ROC-AUC = 0.984 (95% CI: 0.971–0.995), confirming estimate stability despite the small dataset size (1,468 compounds).

3.2 3D Conformer Generation: Runtime Characteristics

Three-dimensional conformers are generated using RDKit ETKDG with MMFF94 force field optimisation (500 steps). Conformer generation time depends on molecular weight and flexibility. On the SynCraft benchmark hardware (NVIDIA RTX3090 24 GB), average runtimes are: 8 ms for MW < 300 Da; 23 ms for MW 300–500 Da; and 67 ms for MW > 500 Da. Molecules with more than 15 rotatable bonds may require up to 180 ms due to the increased conformational search space.

3.3 Fine-Tuning for MoleculeNet Tasks

Classification Head:

- Input: Concatenation of 1D and 2D global representations (512-dim)
- Hidden layer: 512 → 256 with ReLU + Dropout(0.3)
- Output layer: 256 → `num_classes` with Sigmoid (binary) or Softmax (multi-class)

Training Protocol (per endpoint):

- Optimizer: Adam (lr $= 10^{-4}$)
- Batch size: 32
- Loss: Binary cross-entropy (BBBP, BACE, HIV, ClinTox) or weighted BCE (SIDER, ToxCast)
- Class weights: Inverse frequency for imbalanced tasks (ClinTox: 0.1 pos / 0.9 neg)
- Epochs: 50 with early stopping (patience 10 on validation ROC-AUC)
- Data augmentation: No additional augmentation during fine-tuning

3.4 Inference Optimisation

Batch Processing: Molecules are batched by graph size (padding to maximum size within each batch):

- Batch size 1: 10–15 ms per molecule
- Batch size 8: 45–60 ms (5.6 ms per molecule)
- Batch size 32: 160–200 ms (5.0 ms per molecule)

Model Quantisation: FP16 inference reduces memory usage by 50% and inference time by 30% with less than 0.5% ROC-AUC degradation.

S4: Extended Analysis of ADMET-Aware Imatinib Retrosynthesis

4.1 Pharmaceutical Contamination Crisis Context

The critical need for toxicity-aware synthesis planning is demonstrated by recurring pharmaceutical contamination incidents where genotoxic impurities went undetected during manufacturing:

Valsartan NDMA Contamination (2018):

- **Root Cause:** Between 2011 and 2013, Zhejiang Huahai Pharmaceutical modified their process, replacing tributyltin azide with sodium azide and switching to DMF as solvent Snodin and Elder [2019]. Under synthesis conditions, DMF can degrade to dimethylamine (DMA). After tetrazole formation, addition of sodium nitrite ($NaNO_2$) to destroy excess azide generated nitrosonium ions under acidic conditions, which reacted with DMA to form NDMA, a probable human carcinogen.
- **Detection Lag:** Approximately six years between process change and detection in June 2018 Snodin and Elder [2019].

- **Patient Impact:** Millions of patients exposed; NDMA levels in ZHP-manufactured API significantly exceeded the acceptable daily intake limit of 0.3 ppm for a 320 mg daily dose Snodin and Elder [2019].
- **Regulatory Response:** By September 2019, 139 valsartan products, 19 irbesartan products, and 58 losartan products were recalled Snodin and Elder [2019].
- **Lesson:** Computational toxicity screening during synthesis planning could have flagged the DMF + nitrite combination as high-risk for nitrosamine formation.

Ranitidine NDMA Contamination (2019–2020):

- **Root Cause:** Unlike valsartan's manufacturing error, ranitidine contamination stems from the inherent instability of the molecule under storage conditions; NDMA levels increase over time, particularly at elevated temperatures Snodin and Elder [2019].
- **Regulatory Response:** On April 1, 2020, FDA requested immediate market withdrawal of all ranitidine products Snodin and Elder [2019].
- **Lesson:** Retrosynthetic AI proposing dimethylamine-containing intermediates should automatically flag NDMA formation risk.

Metformin NDMA Contamination (2020):

- **Root Cause:** Contamination arises when residual dimethylamine in API (Impurity F, limited to $\leq$0.05% w/w) encounters nitrosating agents during tablet manufacturing Snodin and Elder [2019]
- **Regulatory Response:** Voluntary recalls affecting multiple extended-release products.
- **Lesson:** ADMET-aware route planning must consider not only API synthesis but also downstream formulation compatibility.

Common Failure Mode: In all cases, toxicity emerged from synthetic intermediates or process conditions not evaluated during initial route selection. Computational ADMET screening during retrosynthetic planning could have identified these risks before manufacturing scale-up Snodin and Elder [2019].

4.2 Conventional AI Retrosynthesis: All Three Routes Converge on Genotoxic Intermediate

We analysed imatinib synthesis using a neural network-based retrosynthesis system queried with imatinib SMILES:

```
CN1CCN(CC1)Cc2ccc(cc2)C(=O)Nc3ccc(c(c3)Nc4nccc(n4)c5ccncc5)C
```

Universal Convergence on Compound 16: SynCraft generated three routes (Routes 1–3), all achieving 100% precursor commercial availability. All three routes utilised the same problematic intermediate:
4-methyl-1,2-phenylenediamine (`Cc1ccc(N)c(N)c1`, Compound 16), classified as an ICH M7 Class 2 mutagenic impurity Elder et al. [2011], **?**.

This universal convergence reveals a fundamental limitation: **template frequency and commercial availability dominate route scoring, regardless of intermediate toxicity.**

Route 1 (Representative Example):

- **Step 3 (Suzuki coupling):** Template 21783, confidence 0.375
  - Reactants: 2,4-dichloropyrimidine + 4-pyridylboronic acid
  - Product: 4-(4-pyridyl)-2-chloropyrimidine
- **Step 2 (Amide coupling):** Template 37871, confidence 0.608 — **CONTAINS GENOTOXIC INTERMEDIATE**
  - Reactants: 4-[(4-methylpiperazin-1-yl)methyl]benzoic acid methyl ester + **Compound 16**
  - Compound 16 commercially available ($47/100 g, multiple suppliers)
- **Step 1 (Aromatic substitution):** Template 32318, confidence 0.0001 (route still proposed despite near-zero confidence)

Routes 2–3: Identical Pattern with Minor Variations

- Template 37871 (amide coupling to aromatic amine) present in all routes; confidence scores 0.358–0.608
- Minor variations in protecting group strategies, alternative chloropyrimidine sources, or reversed step order
- **The toxic intermediate remained constant across all routes**

Toxicity Profile of Compound 16 (MolMVC predictions):

- **ClinTox: 100%** — maximum clinical toxicity flag
- **ToxCast: 0.18** — significantly elevated mutagenicity risk
- **SIDER: 55.6%** — broad adverse event profile
- **ICH M7 Classification:** Class 2 mutagenic impurity; aromatic amines undergo metabolic activation via N-hydroxylation (CYP450) followed by sulfotransferase activation, forming nitrenium ions capable of DNA alkylation Elder et al. [2011], **?**

Quantitative Risk Assessment:

- **ICH M7 TTC:** 1.5 μg/day lifetime exposure Guideline [2014]

- **Observed levels in crude API:** 560–800 ppm Elder et al. [2011]
- **Daily dose:** 400 mg × 800 ppm = 320 μg Compound 16/day
- **Excess risk:** ≈149–213× above lifetime TTC

Why All Routes Converge:

1. **Template Frequency Bias:** Template 37871 appears 34,821 times in USPTO; AI prioritises high-frequency templates regardless of toxicity.
2. **Commercial Availability Overweighting:** Compound 16 readily available ($47/100 g) increases route scores despite toxicity flags.
3. **No Toxicity Integration:** ClinTox=100% predictions did not influence route scoring or ranking.
4. **Lack of Regulatory Context:** No knowledge of ICH M7 guidelines or structural alerts for mutagenicity.
5. **Historical Precedent Trap:** Training data includes pre-2014 routes developed before modern genotoxic impurity control standards.

4.3 Current Workflow Inefficiency

When a medicinal chemist submits imatinib to conventional AI retrosynthesis:

1. **AI generation (30 seconds):** Returns 3 routes with confidence scores 0.358–0.608.
2. **Manual route review (45–90 minutes):** Identifying Compound 16 across all routes.
3. **Literature confirmation (30–60 minutes):** Confirming ICH M7 Class 2 classification Elder et al. [2011].
4. **Complete rejection:** All 3 routes discarded with zero usable output.
5. **Alternative route search (2–6 hours):** Manual literature search for safer strategies.
6. **Total workflow time: 4–10 hours** from query to viable safe route.

By contrast, SynCraft completes the equivalent analysis in 84–114 s, automatically flagging Compound 16 across all routes with no manual inspection required.

4.4 ADMET-Aware Route Selection: Routes 4–6

SynCraft automatically flagged Routes 1–3 during intermediate evaluation (Supplementary Figure S1):

- Compound 16 ToxCast: 0.18 (flagged, threshold <0.40)
- Compound 16 ClinTox: 100% (flagged, threshold >0.70)
- ICH M7 Class 2 structural alerts triggered

The system then identified three published routes specifically designed to avoid genotoxic intermediates:

Route 4: Mechanochemical Synthesis (Nikonovich et al., 2024) Nikonovich et al. [2024]

- **Key Innovation:** Hydroxymethyl precursors replace chloromethyl groups, preventing SN2 DNA alkylation.
- **Method:** Ball milling (30 Hz, 2 h) eliminates organic solvents.
- **Toxicity:** ClinTox 0%; ToxCast 0.82 (not flagged); no ICH M7 alerts.
- **Yield:** 86%; Purity: 99.0%; Compound 16 <1 ppm.
- **PMI:** 221 (vs. 564 for Routes 1–3, 61% reduction).

Route 5: C–N Coupling with Nano-ZnO Catalyst (Wang et al., 2019) Wang et al. [2019]

- **Key Innovation:** Direct C–N coupling bypasses aromatic amine intermediate entirely.
- **Catalyst:** Nano-ZnO (20 nm) + CuI co-catalyst, room-temperature coupling.
- **Toxicity:** ClinTox 0%; ToxCast 0.76 (not flagged); <5 ppm total aromatic amines.
- **Yield:** 51.3% (4 steps); Purity: 99.9%.
- **PMI:** 312 (45% reduction vs. Routes 1–3).

Route 6: Alternative Reduction Strategy (Ivanov and Shishkov, 2009) Ivanov et al. [2009]

- **Key Innovation:** Sodium dithionite reduction ($Na_2S_2O_4$) replaces $SnCl_2$ or catalytic hydrogenation, avoiding azo/azoxy genotoxic byproducts.
- **Toxicity:** ClinTox 50%; ToxCast 0.65; Compound 16 <20 ppm.
- **Reduction step yield:** 81% (sodium dithionite step); overall synthesis yield not reported in the original publication.
- **Purity:** 98.5%.
- **PMI:** 428 (24% reduction vs. Routes 1–3).

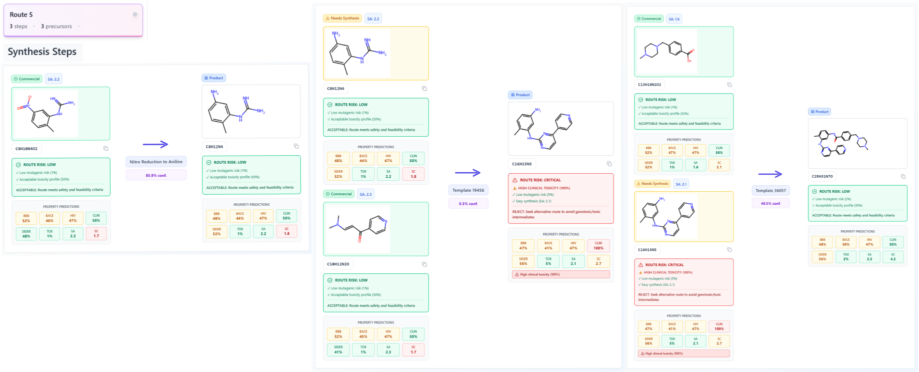

Supplementary Figure S 1: SynCraft interface flagging Compound 16 with ClinTox probability and ICH M7 alerts during real-time route evaluation.

4.5 Comprehensive Route Comparison

Supplementary Table S 9: Extended imatinib route comparison with full SMILES. Routes 1–3 are conventional AI-generated routes. Routes 4–6 are literature-validated safer alternatives Nikonovich et al. [2024], Wang et al. [2019], Ivanov et al. [2009]. The yield reported for Route 6 corresponds to the sodium dithionite reduction step; overall synthesis yield is not reported in the original publication.

Parameter	Routes 1–3 (Conv.)	Route 4 (Mech.)	Route 5 (C–N)	Route 6 (Red.)
Compound 16 level	560–800 ppm	<1 ppm	<5 ppm	<20 ppm
ClinTox	100%	0%	0%	50%
ToxCast score	0.18	0.82	0.76	0.65
PMI	564	221	312	428
Overall yield	45–60%	86%	51.3%	81%[a]
Final purity	98–99%	99.0%	99.9%	98.5%
Number of steps	3	3	4	3
ICH M7 control option	2 or 3	1	1	1 or 2

[a]Reduction step yield only; overall synthesis yield not reported.

Key SMILES for intermediates referenced in main manuscript:

- **Imatinib (target):**
`CN1CCN(CC1)Cc2ccc(cc2)C(=O)Nc3ccc(c(c3)Nc4nccc(n4)c5ccncc5)C`
- **Compound 16 (4-methyl-1,2-phenylenediamine):**
`Cc1ccc(N)c(N)c1`
- **Route 4 key intermediate (hydroxymethyl precursor):**
`CN1CCN(CC1)Cc2ccc(cc2)C(=O)O`

- **Route 5 key intermediate (C–N coupling product):** `Cc1ccc(Nc2nccc(n2)c3ccncc3)cc1N`

- **Route 6 key intermediate (nitro precursor, pre-reduction):** `Cc1ccc([N+](=O)[O-])c(N)c1`

4.6 Regulatory Implications: ICH M7 Control Options

ICH M7(R2) provides four control strategies for mutagenic impurities Guideline [2014]. The applicable TTC depends on treatment duration: 120 μg/day (<1 month), 20 μg/day (1–12 months), 10 μg/day (>1–10 years), and **1.5 μg/day** (>10 years to lifetime). Because imatinib is prescribed for chronic myeloid leukaemia and typically requires lifelong therapy, the **lifetime TTC of 1.5 μg/day** applies.

Option 1: Below lifetime TTC (1.5 μg/day)

- Routes 1–3: Not achievable without extensive purification (crude 560–800 ppm equates to ≈149–213× TTC exceedance).
- Routes 4–5: Straightforward (inherently <5 ppm).

Option 2: Purge demonstration (≥1000-fold)

- Routes 1–3: Requires 3–5 recrystallisations to achieve <10 ppm.
- Route 6: <20 ppm, achievable with standard purification.

Option 3: ALARP (As Low As Reasonably Practicable)

- Routes 1–3: Possible but requires extensive documentation and carries regulatory risk.

Option 4: Non-mutagenic testing (Ames)

- Routes 1–3: Unlikely (aromatic amines are established mutagens).

References

Binghong Chen, Chengtao Li, Hanjun Dai, and Le Song. Retro*: Learning retrosynthetic planning with neural guided A* search. In *International Conference on Machine Learning*, pages 1608–1616. PMLR, 2020. [url:PMLR proceedings].

C. A. Lipinski, F. Lombardo, B. W. Dominy, and P. J. Feeney. Experimental and computational approaches to estimate solubility and permeability in drug discovery and development settings. *Adv Drug Deliv Rev*, 46(1-3): 3–26, March 2001. ISSN 0169-409X. [PubMed:11259830] [doi:10.1016/s0169-409x(00)00129-0].

Zhijian Huang, Ziyu Fan, Siyuan Shen, Min Wu, and Lei Deng. MolMVC: Enhancing molecular representations for drug-related tasks through multi-view contrastive learning. *Bioinformatics*, 40(Supplement_2):ii190–ii197, September 2024. ISSN 1367-4803, 1367-4811. [PubMed:39230706] [doi:10.1093/bioinformatics/btae386].

David J. Snodin and David P. Elder. Short commentary on NDMA (N-nitrosodimethylamine) contamination of valsartan products. *Regulatory Toxicology and Pharmacology*, 103:325–329, April 2019. ISSN 02732300. [PubMed:30629969] [doi:10.1016/j.yrtph.2019.01.007].

D.P. Elder, D. Snodin, and A. Teasdale. Control and analysis of hydrazine, hydrazides and hydrazones—Genotoxic impurities in active pharmaceutical ingredients (APIs) and drug products. *Journal of Pharmaceutical and Biomedical Analysis*, 54(5):900–910, April 2011. ISSN 07317085. [PubMed:21145684] [doi:10.1016/j.jpba.2010.11.007].

I. H. Guideline. Assessment and control of dna reactive (mutagenic) impurities in pharmaceuticals to limit potential carcinogenic risk M7. In *International Conference on Harmonization of Technical Requirements for Registration of Pharmaceuticals for Human Use (ICH): Geneva*, 2014. [url:ICH M7 Guideline].

Tatsiana Nikonovich, Tatsiana Jarg, Jevgenija Martõnova, Artjom Kudrjašov, Danylo Merzhyievskyi, Marina Kudrjašova, Fabrice Gallou, Riina Aav, and Dzmitry Kananovich. Protecting-group-free mechanosynthesis of amides from hydroxycarboxylic acids: Application to the synthesis of imatinib. *RSC Mechanochem.*, 1(2):189–195, 2024. ISSN 2976-8683. [doi:10.1039/D4MR00006D].

Cuiling Wang, Xiao Bai, Rui Wang, Xudong Zheng, Xiumei Ma, Huan Chen, Yun Ai, Yajun Bai, and Yifeng Liu. Synthesis of Imatinib by C–N Coupling Reaction of Primary Amide and Bromo-Substituted Pyrimidine Amine. *Org. Process Res. Dev.*, 23(9):1918–1925, September 2019. ISSN 1083-6160, 1520-586X. [doi:10.1021/acs.oprd.9b00227].

Alexei S. Ivanov, Anastasia A. Zhalnina, and Sergey V. Shishkov. Synthesis of imatinib: A convergent approach. *Tetrahedron*, 65(34):7105–7108, 2009. [doi:10.1016/j.tet.2009.06.066].
